# A Tailored Workflow for Circular RNA Enrichment and Analysis from Human Whole Blood

**DOI:** 10.3390/biom16081149

**Published:** 2026-08-07

**Authors:** Federica Cieri, Valeria Valsecchi, Lorenzo d’Amico di S. Domenico, Raffaele Dubbioso, Marco Salvatore, Lucio Annunziato, Giuseppe Pignataro

**Affiliations:** 1 IRCCS Synlab SDN, Via Galileo Ferraris 144, 80146 Naples, Italy; federica.cieri@synlab.it (F.C.); marco.salvatore@synlab.it (M.S.); lucio.annunziato@gmail.com (L.A.); 2 Division of Pharmacology, Department of Neuroscience, Reproductive and Odontostomatological Sciences, School of Medicine, University of Naples “Federico II”, Via S. Pansini 5, 80131 Naples, Italy; valeria.valsecchi@unina.it (V.V.); damico.lorenzo97@gmail.com (L.d.d.S.D.); 3 Division of Neurology, Department of Neuroscience, Reproductive and Odontostomatological Sciences, School of Medicine, University of Naples “Federico II”, Via S. Pansini 5, 80131 Naples, Italy; raffaele.dubbioso@unina.it

**Keywords:** non-coding RNA, circular RNA, RNA extraction, biomarker discovery, whole blood

## Abstract

CircRNAs are covalently closed ncRNAs originating through back splicing; their expression is finely regulated, displaying specific patterns across different cell types, tissues, and developmental stages. While the molecular functions of circRNAs are not completely elucidated, their regulatory involvement in physiological processes is well established, alongside their dysregulation in several human disorders. These features, together with their higher stability compared to other ncRNAs, make this class of molecules promising theragnostic agents, particularly in biomarker discovery. Accordingly, it is crucial to develop and standardize experimental strategies that improve circRNA analysis, ensuring accurate and effective isolation of these molecules. In biomarker discovery, selecting the appropriate biological matrix is critical; whole blood is often preferred for its accessibility and minimally invasive collection. Because circRNAs are present in human peripheral blood and show promise as disease theragnostic biomarkers, we established a preliminary workflow tailored to isolate and analyze circRNAs from whole blood. The promising effectiveness and robustness of this workflow were demonstrated by qPCR analysis, suggesting highly reproducible detection and reliability in isolating and analyzing circRNAs. This, together with their stability and specific expression profiles, supports the utility of circRNAs in biomarker discovery and advanced circRNA research and contributes to accelerating their future integration into theragnostic applications in clinical settings.

## 1. Introduction

In the last decade, biomarker discovery has become a significant and crucial aspect of medical science [1]. Specific biomarkers show great promise of improving diagnosis (or early diagnosis) and prognosis, especially when combined with other theragnostic modalities. This is particularly essential when dealing with rare, multisystemic, and complex disorders whose diagnostic, monitoring, and treatment strategies are unknown or require significant enhancement. In this regard, the identification of a profile of molecular biomarkers can represent a powerful tool. Interestingly, ncRNAs are an emerging and promising class of molecular biomarkers that could be useful for improving diagnosis and prognosis in several disorders, including neurodegenerative diseases and cancer [2]. Among these, an intriguing subclass is circRNAs, which for many years were dismissed as non-functional by-products of splicing; their biological significance was thus greatly underestimated. Nonetheless, bioinformatics and RNA-seq technologies have shed light on this class of molecules, demonstrating their abundance and suggesting their important role in several biological processes [3]. In particular, circRNAs originate from a back-splicing event leading to a closed head-to-tail RNA molecule [3], characterized by a back-splicing junction (BSJ). Unlike canonical splicing, in which a splice donor and a downstream splice acceptor are covalently bound, in a back-splicing event the splice donor is covalently joined to an upstream splice acceptor, generating a covalently closed molecule lacking a 5′ cap and 3′ polyadenylated tail [3]. Due to this peculiar shape, circRNAs are more stable than other ncRNAs. However, the back splicing is less frequent than the canonical splicing, making circRNAs often less expressed than their linear counterparts [4]. The full range of circRNA functions is still under investigation and has yet to be fully elucidated; however, their crucial role in regulating gene expression through RNA metabolism has been unraveled [5,6,7]. Indeed, circRNAs can modulate gene expression by interacting with transcription factors and RNA-binding proteins (RBPs), and by acting as sponges for microRNAs (miRNAs) [8], thereby sequestering them and reducing their inhibitory effects on target mRNAs [5,6]. Importantly, considering that a single circRNA can sponge more than one miRNA [8], they are able to control the expression of several genes at the same time [9]. For instance, the circRNA derived from the gene *HIPK3* (circ*HIPK3*) has been demonstrated to regulate cell growth by sponging nine different miRNAs [9]. Furthermore, circRNAs can influence the stability of other RNA species, including mRNAs and long non-coding RNAs, and in some cases have been found to undergo cap-independent translation to produce small peptides [10,11,12]. Interestingly, accumulating evidence shows that circRNA expression is altered in disorders characterized by impaired RNA regulation and metabolism, including neurodegenerative diseases and cancers [13,14,15,16,17], suggesting that circRNAs may serve as useful biomarkers in these pathological conditions. This hypothesis is further supported by the high tissue specificity, remarkable stability, and detectable abundance of circRNAs in peripheral blood, features that make them promising candidates for non-invasive biomarker development. Indeed, circRNAs are resistant to exonuclease degradation, show high expression in specific tissues, and can be reliably quantified in blood samples, strengthening their potential for future clinical applications [18,19]. Such potential is particularly compelling for diseases lacking reliable molecular markers, such as ALS (amyotrophic lateral sclerosis), where diagnosis and prognosis remain challenging [20]; indeed, recent studies have identified differentially expressed circRNAs in the blood of ALS patients with high diagnostic accuracy [21]. For instance, specific circRNAs measured in peripheral blood mononuclear cells have shown significant differences between ALS cases and controls and display strong diagnostic potential [21].

Despite this clinical potential, working with circRNAs remains challenging for several aspects, including the low circRNA expression levels compared to their linear counterparts, the still missing availability of a stable reference circular gene, and the lack of a reliable procedure to obtain circRNA from whole blood. Indeed, it should be underlined that the majority of robust workflows adopted for circRNA extraction and enrichment have been performed on cell lines and solid tissues; their direct application on whole blood remains undocumented, and no reference protocols for blood exist. Whole blood is, indeed, a complex matrix, characterized by a high content of RNase, proteins, and enzymatic inhibitors that strongly compromise RNA yield and downstream applications. Therefore, to overcome these methodological complications, currently, blood-based circRNAs are isolated from separated fractions of blood constituents, serum, or PBMC [19,22]. However, despite their efficacy, these approaches, considering the liquid blood fraction only or the cellular component, could limit circRNA recovery, overlooking valuable potential biomarkers.

In light of these premises, to address these limitations and in line with the interest in identifying circRNAs as biomarkers in neurodegenerative diseases, we aimed to establish and validate a robust methodological workflow tailored to isolate and quantify the circRNA pool present in whole blood. Starting from total RNA purification and consequent RNase R treatment, a gold standard procedure in the circRNA field, we first verified the efficacy of the linear RNA enzymatic digestion both through end-point PCR and quantitative real-time PCR, confirming the depletion of a known linear transcript. Then, we focused on the critical issue of selecting a proper reference circRNA for qPCR normalization. Specifically, the stability of three candidate reference circRNAs was assessed. The most stable and reliable in the analyzed cohort was selected as the most promising housekeeping gene for whole blood.

Overall, this approach allows a comprehensive analysis of the entire blood matrix without any fractionation of its constituents, mitigating the risk of losing valuable information.

## 2. Materials and Methods

### 2.1. Whole Blood Collection

Peripheral whole blood samples (volume: ≈ 2 mL) from 8 different healthy males, stratified by age and healthy state, were collected and stored in EDTA tubes (Vacutainer TM K3 EDTA tubes, Becton, Dickinson and Company, Franklin Lakes, NJ, USA; cat. no.: 368857) for 24 h at 4 °C in accordance with our institutional ethical and legal procedures. However, as reported by Wang and Liu [23], the circRNAs in whole blood are stable for at least 24 h.

No specific inclusion or exclusion criteria were applied, as the aim of this study was solely to validate the procedure rather than to perform a clinical evaluation of the study sample.

The study protocol was approved by the local Ethics Committee (Comitato Etico Campania 3) on November 6, 2024 (Protocol No. 297/2024; Project No. PNRR-MCNT2-2023-1237765).

### 2.2. RNA Extraction from Whole Blood

Total RNA extraction was performed using an optimized column-based approach (Quick-RNA Whole Blood kit, Zymo Research Corp, Irvine, CA, USA; cat. no.: R1151).

#### 2.2.1. Sample Stabilization and Proteolysis

Samples were mixed by inverting the tubes 4–5 times. Then, 1 mL of whole blood was incubated for 1 h at room temperature (RT) with DNA/RNA Shield 2× at a 1:1 ratio and proteinase K in a 50:1 ratio. This enzymatic treatment ensures cell lysis, inactivates nucleases and infectious agents, and digests the abundant protein fraction of whole blood.

#### 2.2.2. Pre-Filtration and Nucleic Acids Recovery

Following proteinase K treatment, samples were filtered to recover nucleic acids.

First, 2-propanol 99.9% (Sigma-Aldrich, St. Louis, MO, USA; cat. no: 278475) at a 1:1 ratio was added to each sample. Since the resulting mixture may appear non-homogeneous, it is crucial to briefly vortex it in order to ensure uniformity. Each sample was then transferred into a Zymo-Spin™ IIICG Column and centrifuged (16,000× *g* for 1 min) (Refrigerated Centrifuge Eppendorf 5417R, Eppendorf, Inc., Hamburg, Germany, cat. no: EP-5417R). This step was repeated until the whole mixture was processed.

At this stage, both DNA and RNA were bound to the column matrix and were recovered through RNA Recovery Buffer (200 μL per column) followed by two centrifugations (16,000× *g* for 1 min each).

#### 2.2.3. Isolation and DNase Treatment

To ensure proper RNA binding to the column matrix, 200 μL of 99.9% ethanol (Fisher Chemical TM, Waltham, MA, USA; cat. no.: 10048291) was added to the flow-through. Samples were then mixed well by pipetting until a clear and slightly pink solution was obtained. The mixture was transferred into a Zymo-Spin™ IC Column and centrifuged at 16,000× *g* for 1 min. At this point, the nucleic acids remained bound to the column membrane; thus, a washing step was performed to remove contaminants by adding 400 μL of RNA Wash Buffer to the column. Following this step, an on-column DNase treatment was performed for 30 min at room temperature to digest genomic DNA. Specifically, 40 µL of a digestion mix made of 5 µL of DNase (1 U/µL) and 35 µL of the DNA Digestion Buffer was directly added to the column’s matrix.

#### 2.2.4. Purification and Concentration

Following the on-column DNase treatment, sequential washing steps were performed to remove residual contaminants. First, 400 μL of RNA prep buffer was applied to the column. Following centrifugation, a first wash was carried out by adding 700 μL of RNA wash buffer to the column. A second wash was executed by adding 400 µL of the same buffer and centrifuged at 16,000× *g* for 2 min. Since RNA wash buffer contains ethanol, to ensure its complete removal, an additional empty centrifugation was performed under the same conditions.

Finally, each column was transferred into a sterile, nuclease-free tube to perform RNA concentration and recovery. A volume of 15 µL of DNase/RNase-free water (Zymo Research Corp, Irvine, CA, USA; cat. no.: D4302-5-10) was directly added to the column matrix, and a final centrifugation step was performed. To improve the final yield, the eluate was collected and re-eluted.

#### 2.2.5. RNA Quantification and Quality Assessment

RNA concentrations and purity were assessed using a NanoDrop One spectrophotometer (Thermo Fisher Scientific Inc., Waltham, MA, USA; cat. no: ND-ONE-W). Specifically, 1 µL of total RNA was used to perform quantification, and samples exhibiting A260/280 ratios ≥ 1.8 were chosen for downstream applications.

The RNA yield is sample-dependent. The concentration usually obtained ranges between 100 and 500 ng/µL, corresponding to a total amount of 1.5–7.5 µg of total RNA per 15 µL sample.

### 2.3. CircRNA Enrichment from Total RNA

circRNA enrichment was performed using RNase R (Applied Biological Materials Inc., Richmond, BC, Canada; cat. no.: E049) combined with an optimized column-based approach using Quick-RNA™ Microprep Kit (Zymo Research Corp, Irvine, CA, USA; cat. no: R1050).

#### 2.3.1. RNase R Treatment

Total RNA was treated with the nuclease RNase R to enrich the samples in circRNAs. The 3′– 5′ exonuclease activity of RNase R efficiently digests most of the linear RNAs, enriching the sample in circular RNAs. Moreover, due to putative EDTA residues in the total RNA samples, MgCl_2_ (VWR International by Haasrode, Leuven, Belgium; cat. no.: 5575801) was added to the reaction to improve RNase R activity. Particularly, a 10 µL digestion mix was prepared to treat 1.5 µg of total RNA. The reaction mix contained 1× RNase R reaction buffer, 1 mM MgCl_2_, 7 U of RNase R, and nuclease-free water.

Samples were mixed using a vortex mixer for 5 s and incubated for 40 min at 37 °C using a thermocycler to ensure homogeneous heating (VeritiPro™ Thermal Cycler; Thermo Fisher Scientific Inc., Waltham, MA, USA).

#### 2.3.2. CircRNA Purification

The enrichment in circRNA is followed by a column-based liquids/reaction clean-up to wash out the reaction reagents.

First, RNA Lysis Buffer (Zymo Research Corp, Irvine, CA, USA; cat. no.: R1060) was added to each sample at a 3:1 ratio. Samples were then mixed by vortexing for 5 s. To improve nucleic acid precipitation and column binding, 40 µL of 99.9% ethanol was added to each sample. The resulting solution was thoroughly mixed, transferred into a Zymo-Spin™ IC Column, and centrifuged at 16,000× *g* for 1 min.

Sample purification and concentration were performed by sequential washing steps, each followed by a 16,000× *g* centrifugation step. Specifically, 400 μL of RNA Prep Buffer was applied to the column and centrifuged for 1 min; then, a first wash was carried out by adding 700 μL of RNA Wash Buffer to the column, followed by a second wash with 400 µL of the same buffer and 2 min centrifugation at 16,000× *g*. As previously mentioned, to ensure complete ethanol removal, an additional 2 min dry centrifugation step was performed. Finally, as previously described, a double elution in 15 µL of DNase/RNase-Free Water was performed by adding it directly to the column matrix.

#### 2.3.3. CircRNA Quantification

CircRNA concentration was assessed using a NanoDrop One spectrophotometer (Thermo Fisher Scientific Inc., Waltham, MA, USA; cat. no: ND-ONE-W). Specifically, 1 µL of enriched eluate was used to perform quantification.

Since circRNAs represent only a small fraction of the total RNA, the circRNA yield is strongly reduced compared to its unenriched counterpart. The obtained yield is among 10–20 ng/µL. Thus, an amount of 110–280 ng of RNA enriched in circRNAs is expected.

### 2.4. cDNA Synthesis

Two hundred nanograms of circRNA or total RNA was reverse-transcribed using random primers and MultiScribe Reverse Transcriptase Kit (Thermofisher Scientific Baltics, Vilnius, Lithuania; cat. no: 4319983/4311235) following the manufacturer’s protocol. The reaction was prepared in 20 µL, yielding a nominal cDNA concentration profile ranging from 10 to 15 ng/µL. The cDNA samples were stored at −20 °C until downstream applications.

### 2.5. Divergent Primer Design

hsa_circ_0000284 and hsa_circ_0000471 primers were designed using Molecular Biology Suite (Benchling, Biology Software, 2025. Retrieved from https://benchling.com, accessed on 4 August 2026) based on the circRNA target sequence obtained from Circbank [24] and circBase databases [25]. They are characterized by a divergent orientation, flanking or overlapping the back-splicing junction (BSJ), to ensure specific amplification of the circular targets.

All primers, listed in Table 1, operate at an annealing temperature of 60 °C, and the amplicon size is in the range of 115–160 bp.

### 2.6. Qualitative and Quantitative Polymerase Chain Reaction (PCR)

Five nanograms of cDNA was amplified in a 10 µL reaction mix for qualitative PCR analysis. YourSial Taq HS Mix (Sial S.r.l, Rome, Italy; cat. no: SIAL-yHSTmix) 1 × as final concentration, 500 nM of each specific primer (synthesized by Eurofins Genomics, Ebersberg, Germany), and nuclease-free water up to the final volume were mixed. The results were checked on 1.5% agarose gel (SeaKem^®^ LE Agarose, Lonza, Rockland, ME, USA; cat. no: 50005) prepared in 1× Tris/Acetic Acid/EDTA (TAE). Safe-Green™ (Applied Biological Materials Inc., Richmond, BC, Canada; cat. no.: G108-G) was adopted as loading dye to visualize the amplicon. Fragment sizes were determined using Gel-Pilot DNA Molecular Weight 100 bp (QIAGEN GmbH, Hilden, Germany; cat. no. 239035). Gel was detected with GelDoc Go (Bio-Rad Laboratories, Inc., Hercules, CA, USA). The optical density of the obtained amplicons was determined with Quantity One^®^ 1-D Analysis Software Version 4.6.6 (Bio-Rad Laboratories, Inc., Hercules, CA, USA), using the 400 bp ladder band as reference.

For quantitative PCR (qPCR), 5 ng of cDNA was amplified. A 10 µL reaction mix was prepared by mixing Sensi Fast Syber Mix (SensiFAST™ SYBR^®^ No-ROX Kit, Meridian Bioscience, Inc., Cincinnati, OH; cat. no: BIO-98050) at 1 × final concentration, 200 nM of forward and reverse primers specific for the desired targets, and nuclease-free water up to the final volume. Samples were amplified with a CFX96 Touch Real-Time PCR Detection System (Bio-Rad Laboratories, Inc., Hercules, CA, USA). The results were finally analyzed through CFX Maestro Software Version 5.3.022.1030 (Bio-Rad Laboratories, Inc., Hercules, CA, USA).

### 2.7. Statistical Analysis

GraphPad Prism (version 10.3.0) was adopted to perform statistical analysis and graphs. qPCR data derived from cDNAs of independent biological replicates (n = 8) were analyzed. Particularly, for each individual tested, cDNA of total RNA (RNase R−) and cDNA of RNA enriched in circRNAs (RNase R+) were obtained. SD was calculated through Descriptive Statistics. The analyzed data consisted of paired observations from independent biological replicates. Thus, a non-parametric Wilcoxon matched-pairs signed rank test was employed to compare the Ct values of untreated and treated groups and determine their putative sensitivity to RNase R. Data were shown using symbols and a line graph to visualize each biological replicate (*p* > 0.05).

### 2.8. Software and Databases

Target circRNA sequences were identified and retrieved using Circbank [24] and circBase [25] databases. Primers designed in this study were generated using Molecular Biology Suite (Benchling, Biology Software, 2025. Retrieved from https://benchling.com, accessed on 4 August 2026) based on the Primer 3 algorithm, as a primer design tool. Gel images were acquired using Image Lab Touch Software 3.0.0.07 (Bio-Rad Laboratories, Inc., Hercules, CA, USA). Densitometry analysis was performed using Quantity One^®^ 1-D Analysis Software Version 4.6.6 (Bio-Rad Laboratories, Inc., Hercules, CA, USA). Quantitative PCR results were analyzed using CFX Maestro Software Version 5.3.022.1030 (Bio-Rad Laboratories, Inc., Hercules, CA, USA), and GraphPad Prism Version 10.3.0 was adopted as statistical software.

## 3. Results

In this study, available standard tools were adapted and refined in order to establish a preliminary workflow tailored to obtain circRNAs from whole blood without any prior separation of its constituents. By avoiding the common fractionation of blood constituents, such as plasma, serum, or PBMC, this method provides a global look at the whole blood matrix, minimizing the loss of valuable putative data. Total RNA was extracted from peripheral whole blood from eight different healthy subjects. RNA yield and samples’ quality and purity were assessed through spectrophotometry prior to downstream applications. Then, each sample was treated with RNase R to enrich it in circRNAs (circRNA yield of 16.8 ± 4 ng/µL in total RNA yield of 270.4 ± 123 ng/µL) (Table 2).

### 3.1. RNase R Treatment Is Specific for Linear Transcripts

**Figure 1:**
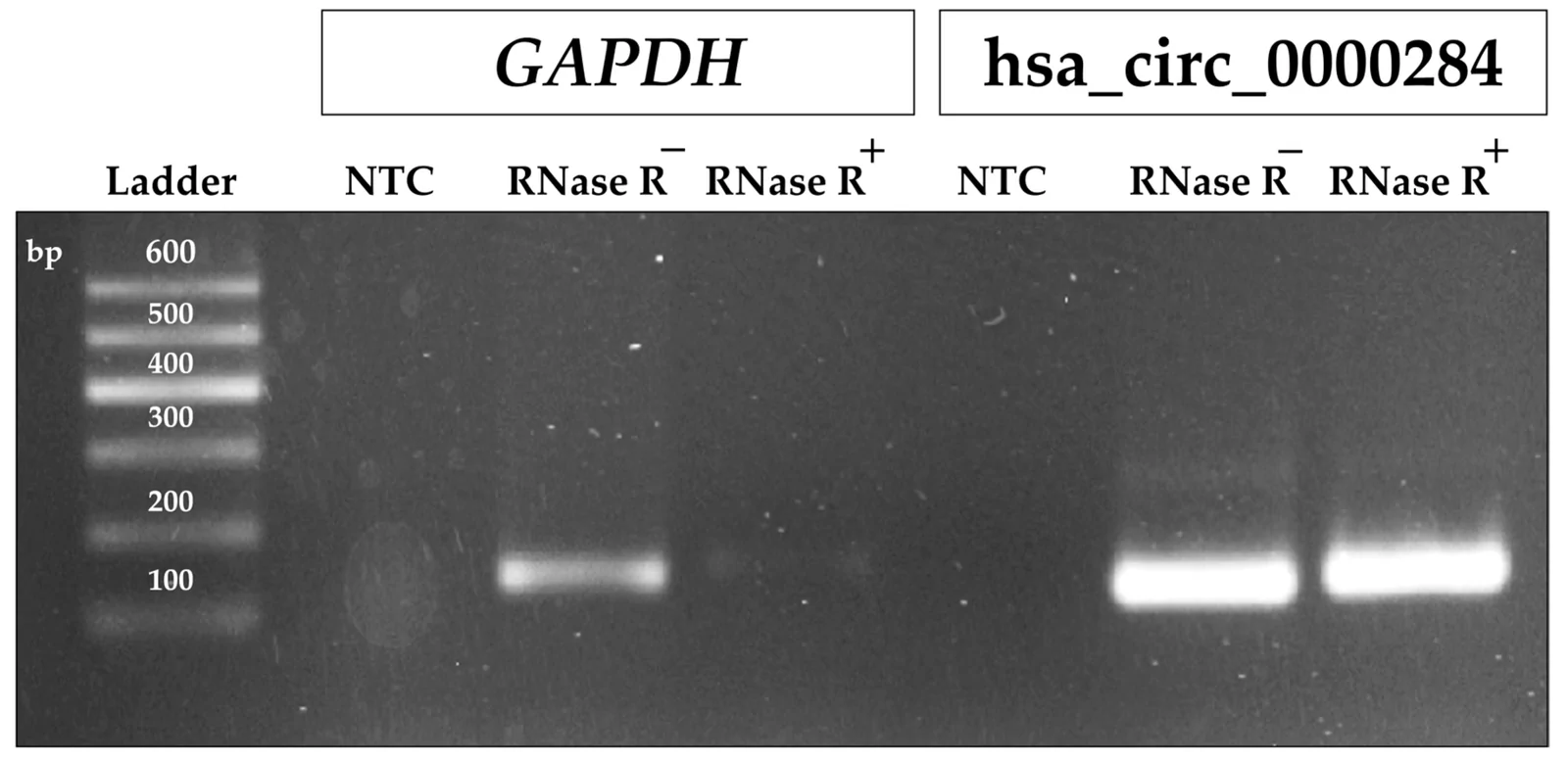
Qualitative PCR on untreated samples and RNase R-treated samples. The figure shows a representative gel of the PCR results on cDNA obtained from samples untreated (RNase R−and treated (RNase R+) with RNase R. The same sample was tested with *GAPDH* (122 pb) and hsa_circ_0000284 (126 pb). In RNase R- samples, both *GAPDH* and hsa_circ_000284 were detectable; in RNase R+ samples, only hsa_circ_000284 was detectable. NTC stands for no template control; it is a control reaction performed without cDNA template to check for contamination of PCR reagents. Ladder is Gel Pilot DNA Molecular Weight 100 pb.

To assess RNase R-treatment efficacy, an end-point PCR to amplify *GAPDH* (Glyceraldehyde 3-phosphate dehydrogenase) was performed both in RNase R-treated and untreated samples. Due to RNase R nuclease activity, a linear transcript, such as *GAPDH*, should be poorly expressed in samples enriched in circRNA compared to their un-enriched counterpart. Interestingly, a barely detectable amplification of *GAPDH* was observed in samples treated with RNase R compared to the untreated ones (Figure 1), demonstrating that the RNase R treatment has been effective. Indeed, a densitometric analysis detected a signal for the *GAPDH* amplicon 16-fold higher in RNase R-untreated samples compared to the one obtained with enriched RNA samples.

Additionally, to evaluate whether the digestion treatment was specific for linear RNAs, a circRNA was also tested (hsa_circ_0000284) (Figure 1). As expected, hsa_circ_0000284 was amplified in both RNase R-treated and untreated samples, demonstrating that the digestion is specific for linear RNAs and does not affect the circRNA species. Indeed, the densitometric analysis for the hsa_circ_0000284 amplicon gave a comparable signal both in RNase R-treated and untreated samples.

### 3.2. Quantitative Analysis Confirms RNase R Specificity and Efficiency

**Figure 2:**
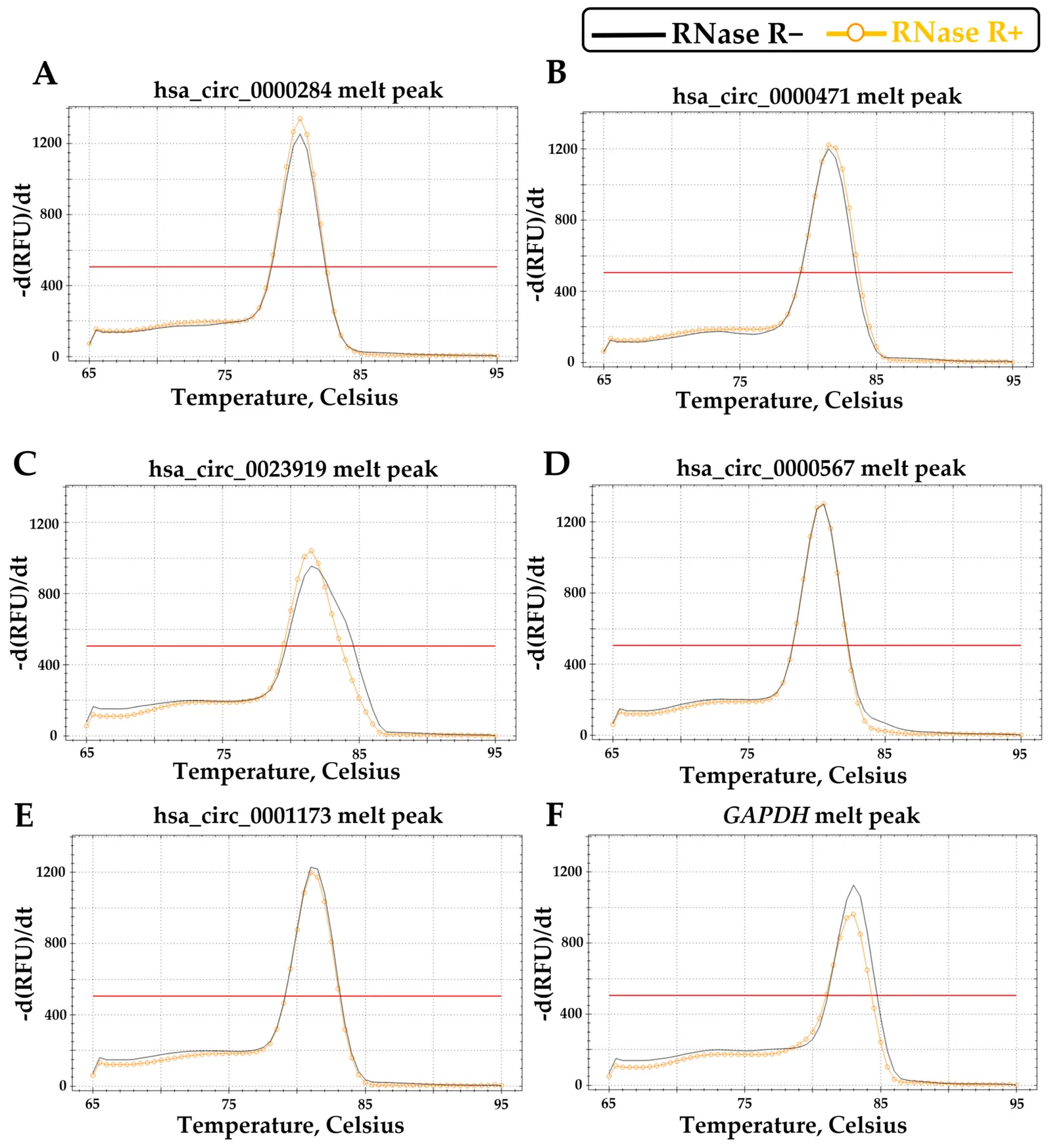
qPCR on samples treated and untreated with RNase R. Representative melting curves obtained for each selected circRNA and *GAPDH*. Black curves are data related to samples not digested with RNase R (RNase R−), orange curves with circles are related to samples treated with RNase R (RNase R+). Every peak in the melting curve indicates a PCR product; the red line is the melt peak threshold. (**A**–**F**): Melting peaks show the rate of change in the fluorescence signal over time (dRFU/dt) as a function of the temperature. Eight samples were analyzed. All graphs were obtained from CFX Maestro Software Version 5.3.022.1030.

To further strengthen our previous results on RNase R treatment effectiveness, a quantitative analysis through qPCR was performed. In particular, the expression levels of *GAPDH* and five circRNAs (hsa_circ_0000284, hsa_circ_0000471, hsa_circ_0023919, hsa_circ_0000567 and hsa_circ_0001173), that have been previously demonstrated to be expressed in whole blood [18], were assessed. The amplification curves, the melting temperature (Supplementary Table S1) and peaks, and Ct values were analyzed. In particular, in both RNase R+ and RNase R− samples, the melting peak analyses of each circRNA tested (Figure 2A–E) and *GAPDH* (Figure 2F) showed a single and well-defined peak, confirming the specificity of the amplification and the robustness of divergent primers designed for circRNA.

Interestingly, the *GAPDH* melting peak (Figure 2F) has a lower height in the RNase R+ samples (Figure 2F, orange peak) compared to the RNase R− ones, suggesting a lower starting template amount of *GAPDH* levels in RNase R-treated samples, possibly due to the efficient digestion of the linear RNA species. This was also confirmed by Ct values (Supplementary Table S2). Indeed, *GAPDH* Ct values were higher (around 30) in samples enriched in circRNA compared to the unenriched ones, confirming that *GAPDH* expression is strongly reduced in RNase R+ samples as linear RNAs are mostly degraded and that the enzymatic treatment was efficient. Specifically, the ΔCt between the same sample RNase R+ and RNase R− was calculated (Table 3; Supplementary Table S2; Supplementary Figure S1). The obtained values were around four cycles, suggesting that linear transcript expression in RNase R+ samples is 16-fold (2^4^) lower than in the unenriched samples (RNase R−) [27].

**Figure 3:**
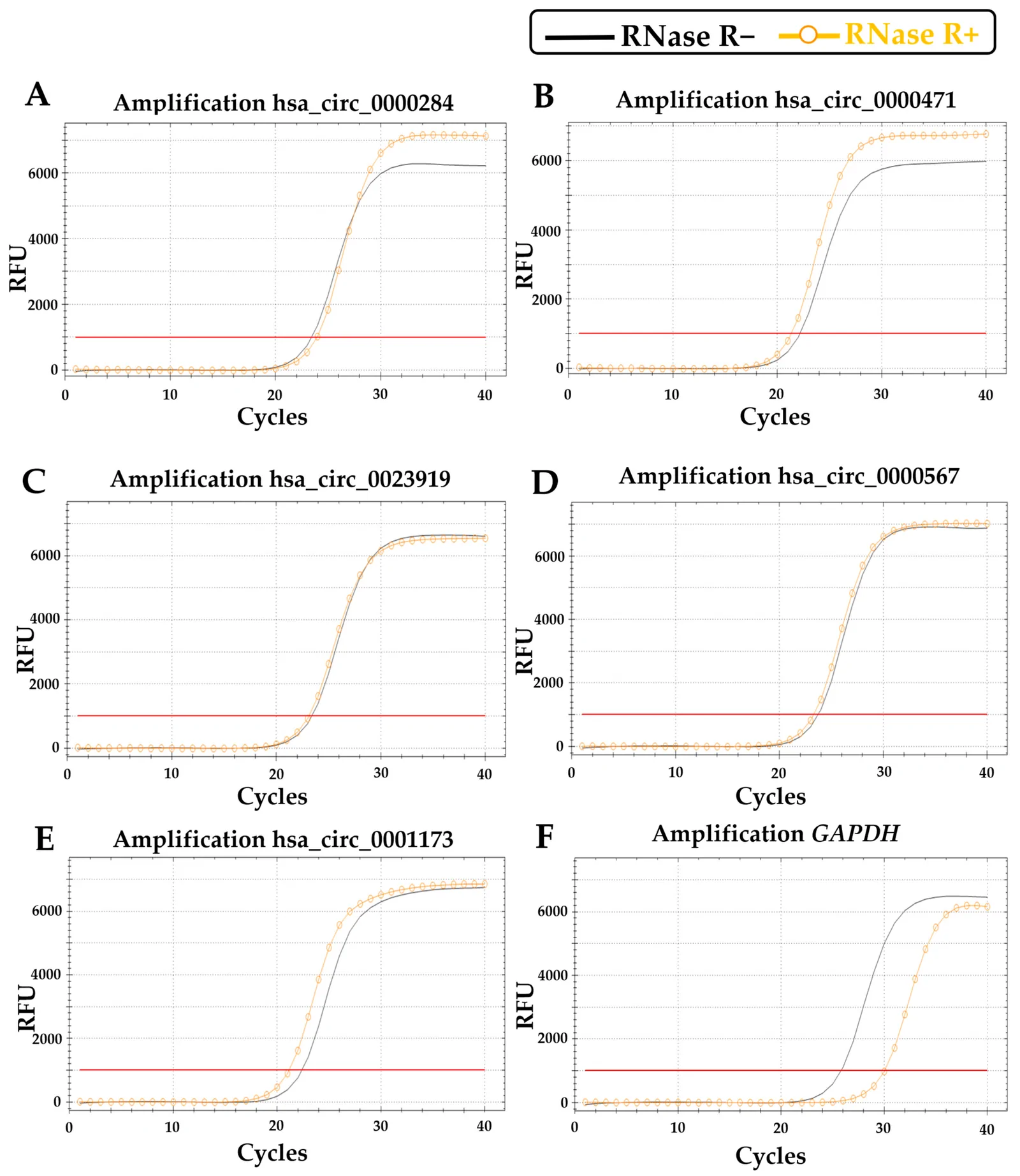
Amplification curves of samples treated and untreated with RNase R. Representative amplification curves obtained for each selected circRNA and *GAPDH*. Black curves are data related to samples not digested with RNase R (RNase R−), and orange curves with circles are samples treated with RNase R (RNase R+). (**A**–**F**): Amplification curves show the Relative Fluorescence Unit (RFU) as a function of the number of cycles (Ct); the red line is the amplification threshold. 8 samples were analyzed. All graphs were obtained from CFX Maestro Software Version 5.3.022.1030.

Conversely, the representative amplification curves related to circRNA targets (Figure 3A–F) confirm circRNA structural stability and their overall resistance to RNase R. Indeed, Ct values for circular targets remain stable in RNase R+ samples (orange curves with circles) compared to the RNase R− ones (black curves) (Table 3), confirming that RNase R does not affect circular RNA species.

### 3.3. hsa_circ_0000471 Is a Promising Reference Gene in Whole Blood

Finally, for a more accurate evaluation of circRNA expression levels, we performed tests to select a suitable circRNA as reference gene for normalization in whole blood, since qPCR remains the standard approach and the establishment of a reliable reference gene is one of the major challenges in the circRNA field. circRNAs and linear RNAs represent different classes of molecules. Moreover, due to the sensitivity of linear transcripts to RNase R treatment—a standard and crucial procedure in this field—they may not represent the most suitable reference genes for circular targets.

Following a transcriptomic analysis, Zhong et al. [28] identified hsa_circ_0000284, hsa_circ_0000471, and hsa_circ_0000567 as promising circRNA reference genes, and selected hsa_circ_0000284 and hsa_circ_0000471 as more suitable in cell lines. Considering that these three circRNAs are expressed in whole blood [19], we hypothesized they could be putative candidates to be validated in this matrix. To confirm our hypothesis, we evaluated hsa_circ_0000284, hsa_circ_0000471 and hsa_circ_0000567 Ct values across all samples before and after RNase R treatment.

**Figure 4:**
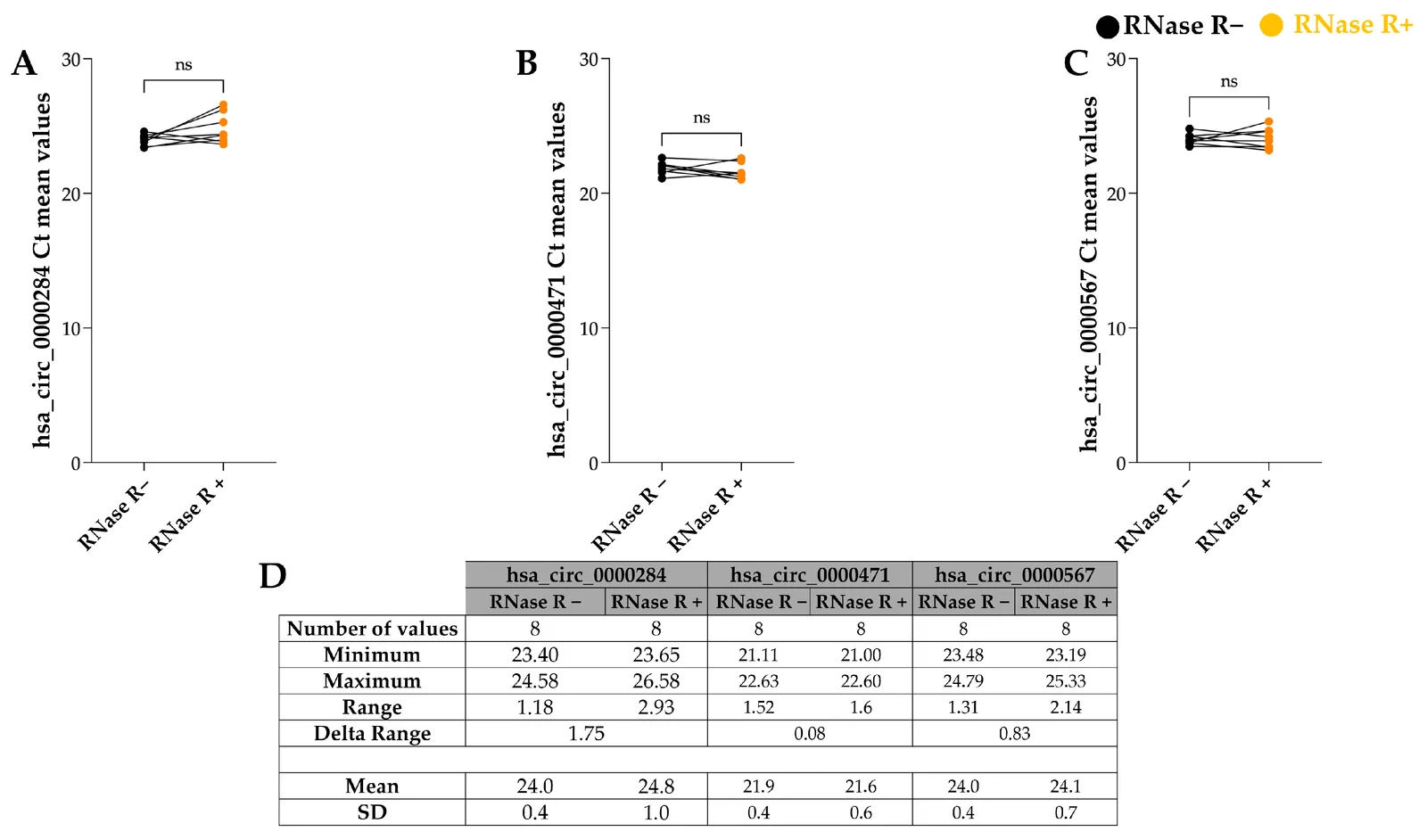
Putative reference circRNA Ct values: Ct values of candidate reference circRNA and descriptive statistics. (**A**–**C**). Symbols and line graphs show the Ct mean obtained for each biological sample untreated (RNase R−, black dots) and treated with RNase R (RNase R+, orange dots). Each symbol represents a sample. Statistical analysis was performed through a non-parametric Wilcoxon matched-pairs signed-rank test; ns, not significant. (**D**) reports the descriptive statistics.

Following an overall Ct analysis, all three candidates appeared stable across different samples (Table 3), exhibiting technical and biological reproducibility and no statistically significant fluctuation across RNase R-treated and untreated samples (Figure 4).

However, by analyzing the range of Ct values recorded for each sample within each condition tested, hsa_circ_0000284 and hsa_circ_0000567 showed higher sample to sample fluctuation in RNase R cohort compared to hsa_circ_0000471 (range of hsa_circ_0000284: 2.93; hsa_circ_0000471: 1.60; hsa_circ_0000567: 2.14). Indeed, assessing a Delta Range, defined as the shift in “the maximum-to-minimum Ct range” obtained between treated and untreated states, these two circular RNAs showed higher sensitivity to RNase R. Specifically, the delta range of hsa_circ_0000284 was 1.75, and hsa_circ_0000567 was 0.83 versus hsa_circ_0000471, which was only 0.08 (Figure 4D).

Therefore, hsa_circ_0000471 showed a higher stability to the enzymatic treatment and a lower fluctuation in the analyzed cohort, highlighting its potential as a reference circRNA in whole blood.

On the other hand, *GAPDH* Ct values were statistically significantly higher in RNase R-treated samples, reinforcing previous data and confirming that it is not a suitable housekeeping gene for circRNA quantification (Supplementary Table S2; Supplementary Figure S1).

## 4. Discussion

The present study illustrates for the first time an efficient and customized strategy to enrich and isolate circRNAs from whole blood, allowing the analysis of the entire biological matrix without fractioning it. This methodology addresses for the first time the challenges occurring during RNA extraction from whole blood, providing a workflow from sample preparation to downstream application and reducing the risk of losing potentially valuable and highly specific new targets.

**Figure 5:**
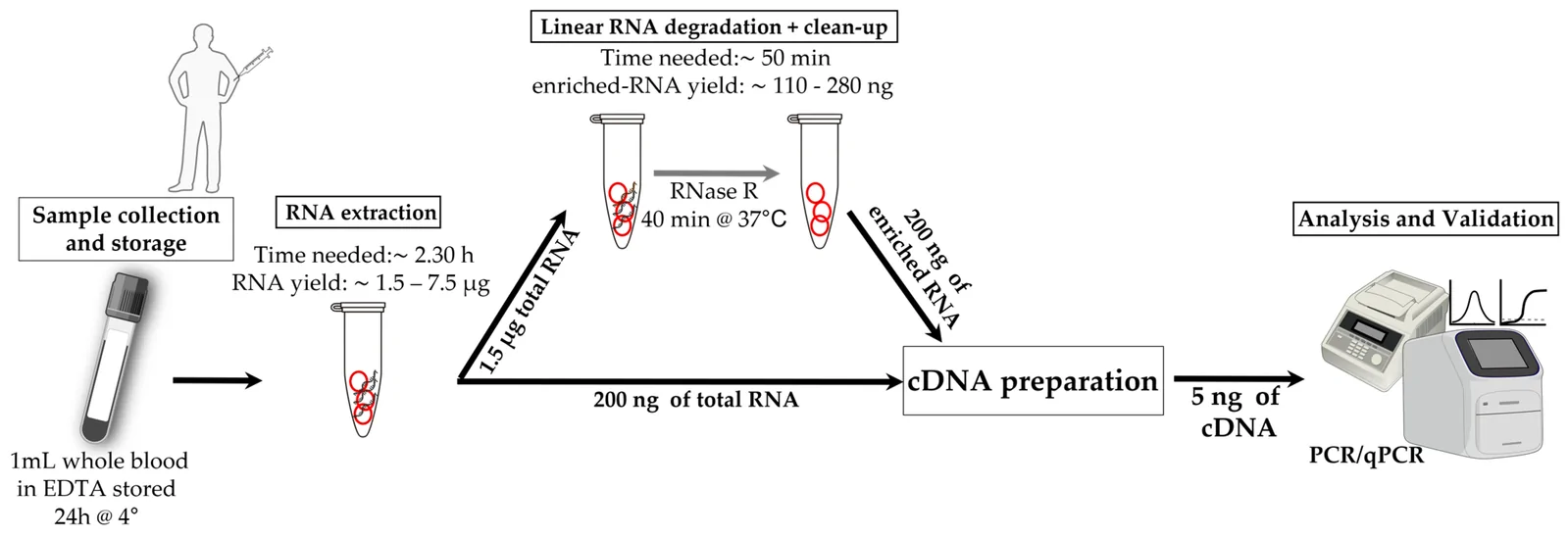
Schematic protocol workflow: 1 mL of whole blood, stored in EDTA tubes for 24 h at 4 °C, was used to extract total RNA. Next, 1.5 μg of RNA was treated with RNase R to enrich samples in circRNA and then cleaned up. The final yield is around 110–280 ng. The same amount of circular-enriched RNA and total RNA was then reverse transcribed. Five ng of cDNA was used for downstream applications, PCR and qPCR.

In particular, we established a preliminary workflow (Figure 5) to extract circRNAs from whole blood without any fractionation of its constituents. By avoiding the common fractionation of blood constituents, such as plasma, serum, or PBMC, this method tailored to whole blood provides a global look at this matrix, minimizing the loss of valuable putative data.

The primary challenge that occurs during RNA extraction is the rehomogenization of EDTA-blood samples after 4 °C storage. During storage, cells undergo sedimentation; thus, inadequate mixing could strongly compromise RNA yield. To address this, samples should be properly resuspended by gentle tube inversion immediately before lysis, as underlined in the method section of the present paper.

Furthermore, whole blood, due to its high protein content, is rich in PCR inhibitors such as hemoglobin and immunoglobulin G [29]. This peculiar aspect was solved by introducing a long digestion step with proteinase K at the very beginning of the total RNA extraction phase, as shown in the method section. Protein contamination was assessed by a spectrophotometric analysis monitoring the A260/280 ratios (acceptable range from 1.9 to 2.1 in agreement with standard RNA extraction procedures (Quick-RNA Whole Blood kit, Zymo Research Corp; Table 1), confirming the effectiveness of the enzymatic digestion.

Besides PCR inhibitors, the whole blood matrix has a high content of RNases that could strongly compromise total RNA yield. To mitigate this and preserve its integrity, RNA extraction was performed under strict RNase-free conditions and immediately processed and stored at −80 °C.

Another crucial aspect to consider is that RNase R is a magnesium-dependent exonuclease that requires Mg^2+^ to work properly. Whole blood samples are commonly collected in EDTA tubes; however, this chelating agent can sequester metal cofactors, inhibiting RNase activity when its residues in the RNA extract are too high, compromising circRNA enrichment. In the present paper, we optimized this aspect, supplementing the digestion reaction with MgCl_2_ (1 mM), as described.

Finally, since RNase R efficiency can depend on the RNA secondary structures, partial digestion of the linear transcripts can occasionally occur. Thus, to ensure linear transcript digestion without imparing circRNA yield, forty minutes RNase R incubation was chosen. At this time point, the control linear transcript (*GAPDH*) was successfully degraded (Figure 1; Supplementary Table S1) without compromising circRNA yield.

Thus, it is strongly recommended to use a linear RNA target as a control in the downstream application. However, if incomplete digestion is frequently observed, then it is possible to extend the RNase incubation time to not exceed 1 h of incubation.

The potential robustness and reliability of the established workflow have been tested through qualitative and quantitative PCR. An initial qualitative test, confirmed by densitometry analysis, demonstrates that the RNase R treatment worked effectively on a linear RNA transcript, that differently from circRNA, has a 3′-free-end available for exonuclease activity. Indeed, in cDNAs derived from total RNA, both a linear and a circular transcript were amplified, while in samples enriched for circRNAs, only circular targets were detectable (Figure 1). In line with our results, Abe et al. [30], who tested circRNA migration on a standard agarose system post PCR, observed that a linear target is undetectable post-RNase R treatment, corroborating our data and supporting the consistency of the presented approach.

A similar result was also obtained through quantitative analysis. Indeed, while Ct values for *GAPDH* show a four-cycle delay in RNase R-treated samples (Supplementary Table S2, Supplementary Figure S1), Ct values for the circRNA target remain consistent across treated and untreated conditions (Table 3). Thus, proving the consistent efficiency of the treatment and reinforces the gold standard of RNase R treatment to obtain an optimal relative expression profile.

An additional key point of the presented work regards the selection of a circular housekeeping gene to normalize circular targets through qPCR, which remains the standard and most accessible approach adopted. As a consequence, the establishment of a reliable and biologically robust normalization strategy remains one of the major challenges in the circRNA field. Several studies use linear housekeeping genes for normalization [14,21], incurring the risk of introducing bias in the circRNA quantification, as conventional linear reference genes are biologically different from circular transcripts, besides being digested by RNase R. Thus, through the selection of a circRNA normalizer, our approach ensures a more biologically robust framework for circRNA expression analysis. This is because both target genes and reference genes exhibit the same response to enzymatic digestion and preserve the same ratio even after RNase R treatment.

Particularly, hsa_circ_0000284, hsa_circ_0000471, and hsa_circ_0000567 were evaluated as promising reference circRNAs as previously reported by Zhong et al. [28]. CircRNA expression can be highly context-dependent and heavily influenced by pathological backgrounds; therefore, their stability, it is crucial. An unstable reference gene might cause significant technical artifacts, misinterpretation, and miscalculation of biological results. This is critical when the RNase R treatment is performed.

In this regard, hsa_circ_0000284, hsa_circ_0000471, and hsa_circ_0000567 were evaluated across all samples before and after RNase R treatment. Interestingly, our results showed that among the tested circRNAs, hsa_circ_0000471 is the most stable across the analyzed cohort, specifically within the RNase R+ subgroup. This highlights hsa_circ_0000471 as the most reliable candidate reference circRNA, thereby providing a validated and reproducible housekeeping for circRNA whole blood profiling.

The main limitation of the paper is represented by the sample size, as it includes only eight participants, and the use of NanoDrop as the main quantification method. In addition, we acknowledge that maintaining a similar Ct does not necessarily imply that the absolute quantity of each circRNA has been completely preserved.

Altogether, these findings contribute to improving circRNA analysis and quantification by paving the way for a robust technical advancement that enhances circRNA profiling and strengthens biomarker discovery and clinical screening strategies.

## 5. Conclusions

In conclusion, the workflow presented in this study offers a strategy to obtain circRNAs from whole blood with high reliability potential. This approach could significantly contribute to future advancement in circRNAs, particularly regarding their prospective utility as disease biomarkers. This aspect is a crucial and timely topic in medical science, as it can contribute to enhancing future theragnostic strategies, thus ameliorating patients’ healthcare. Indeed, it is now clear that the role of non-coding RNAs in regulating gene expression and their value in health and human diseases, and focusing on non-coding RNAs, such as circRNAs, could be a powerful and innovative research strategy.

## Figures and Tables

**Table 1:** Primer list.

Primer Name	Primer Sequences 5′→3′	Reference
hsa_circ_0000284	Fwd: CTGTTCGGCAGCCTTACAGGG Rev: GGGTAGACCAAGACTTGTGAGGC	Designed in the present study
hsa_circ_0000471	Fwd: CCTCCTCCACAGGGGAGACAG Rev: TGGAGATCTTCCCTGATCGATAGC	Designed in the present study
hsa_circ_0023919	Fwd: ATTTGCAGCAGCCAACTTTT Rev: CCTGCTTGCAGCTGTAGAATC	[21]
hsa_circ_0000567	Fwd: AAACACAGCTCGACAGTACGC Rev: TCCTTTGGTGACACAGTTGC	[22]
hsa_circ_0001173	Fwd: TGCAAGGTGAAGTTCAGAGG Rev: TCTGCTGGCAATTCAAACAC	[22]
*GAPDH*	Fwd: ATGTTTGTGATGGGTGTGAA Rev: ATGCCAAAGTTGTCATGGAT	[26]

**Table 2:** Spectrophotometric analysis of total RNA.

	Total RNA Yield	A260/280	circRNA Yield	A260/280
1	340.50 ng/µL	2.02	13.30 ng/µL	1.83
2	194.50 ng/µL	1.99	14.20 ng/µL	1.81
3	200.00 ng/µL	2.01	10.00 ng/µL	1.83
4	102.00 ng/µL	1.96	20.30 ng/µL	1.86
5	304.30 ng/µL	2.03	17.90 ng/µL	1.94
6	466.20 ng/µL	2.07	20.60 ng/µL	1.89
7	285.50 ng/µL	2.04	21.20 ng/µL	1.80
8	107.30 ng/µL	2.02	22.2 ng/µL	1.97

Concentrations and A260/280 values for each sample tested. The table shows the total RNA and circRNA yields and A260/280 ratios obtained for each sample. Ratios range between 1.80 and 2.07, suggesting that samples are free from protein contamination. Data were obtained using a NanoDrop One spectrophotometer (Thermo Fisher Scientific Inc., Waltham, MA, USA; cat. no: ND-ONE-W).

**Table 3:** Target Ct mean values.

Targets	RNase R−	RNase R+	∆Ct [(RNase R+ Ct Mean) − (RNase R− Ct Mean)]
	Ct Mean	Ct Mean	
hsa_circ_0000284	24.0 ± 0.4	24.8 ± 1.0	0.8
hsa_circ_0000471	21.9 ± 0.4	21.6 ± 0.6	−0.3
hsa_circ_0023919	23.2 ± 0.4	23.4 ± 0.7	0.2
hsa_circ_0000567	24.0 ± 0.4	24.1 ± 0.7	0.1
hsa_circ_0001173	22.5 ± 0.6	21.7 ± 0.7	−0.8
*GAPDH*	26.0 ± 0.8	29.7 ± 0.8	3.3

Ct values and ΔCt. The table shows the means of Ct values ± standard deviation obtained from 8 independent biological replicates for each target tested both in samples untreated and treated with RNase R; the difference in Ct values between RNase R+ and RNase R− samples. While ΔCt in circRNA targets remains stable, ΔCt in *GAPDH* is higher than 3 cycles, suggesting the enzymatic specificity for linear transcripts and a strong reduction in *GAPDH* in samples enriched in circRNAs.

## Data Availability

The original contributions presented in this study are included in the article/Supplementary Material. Further inquiries can be directed to the corresponding author.

## Supplementary Materials

The following supporting information can be downloaded at: https://www.mdpi.com/article/10.3390/biom16081149/s1, Table S1: Melting temperatures; Table S2: *GAPDH* Ct values and ΔCt; Figure S1: *GAPDH* Ct values.

## Abbreviations

The following abbreviations are used in this manuscript:

CircRNAs	Circular RNA
ncRNAs	Non-coding RNAs
qPCR	Quantitative polymerase chain reaction
RNAseq	RNA sequencing
BSJ	Back splicing junction
RBPs	RNA-binding proteins
miRNAs	Micro RNA
mRNAs	Messenger RNA
ALS	Amyotrophic lateral sclerosis
RNase	Ribonuclease
PCR	Polymerase chain reaction
EDTA	Ethylene–diamine–tetraacetic acid
PBMC	Peripheral blood mononuclear cells
RT	Real temperature
DNA	Deoxyribonucleic acid
Bp	Base pair
cDNA	Complementary DNA
SD	Standard deviation
*GAPDH*	Glycerinaldehyde-3-phosphate dehydrogenase
NTC	No-template control
Ct	Cycle threshold
