# Supplementary material for: A Tailored Workflow for Circular RNA Enrichment and Analysis from Human Whole Blood
Source: Biomolecules. 2026 Aug 7;16(8):1149. doi: 10.3390/biom16081149 (PMC13510710; doi:10.3390/biom16081149)
Supplement: Supplementary file 1 [file biomolecules-16-01149-s001.zip › biomolecules-4458255-supplementary.pdf]

Supplementary Materials

Supplementary Table S1. Melting temperatures.

Targets	Melting Temperatures	
	RNase R−	RNase R+
hsa_circ_0000284	80.5°C	80.5°C
hsa_circ_0000471	81.5°C	81.5°C
hsa_circ_0023919	81.5°C	81.5°C
hsa_circ_0000567	80.2°C	80.2°C
hsa_circ_0001173	81.2°C	81.2°C
GAPDH	83.0°C	83.0°C

Supplementary Table S2. *GAPDH* Ct values and ΔCt.

	RNase R−	RNase R+	ΔCt
**1**	26.05	29.86	3.81
**2**	25.96	30.23	4.27
**3**	25.91	29.58	3.67
**4**	26.26	30.07	3.81
**5**	26.97	30.54	3.57
**6**	26.24	29.36	3.13
**7**	24.41	28.21	3.81
8	26.80	30.07	3.27

Supplementary Table S2. *GAPDH* Ct values and ΔCt. The table shows the Ct values per different samples untreated and treated with RNase R, and the difference in Ct values between the RNase R+ and RNase R− samples. ΔCt values are higher than 3 cycles, suggesting a strong reduction in *GAPDH* in samples enriched in circRNAs. Graphs were obtained from CFX Maestro Software Version 5.3.022.1030.

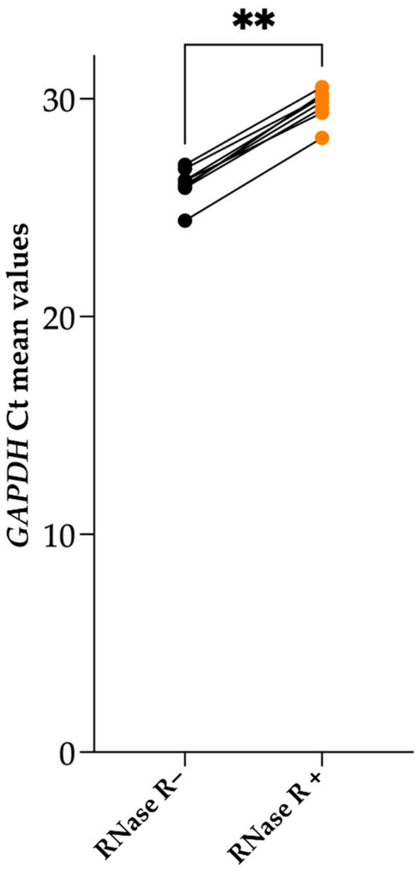

Supplementary Figure S1. *GAPDH* Ct values: Symbols and line graphs show the Ct mean obtained for each biological sample untreated (RNase R−, black dots) and treated with RNase R (RNase R+, orange dots). Each symbol represents a sample. Statistical analysis was performed through a non-parametric Wilcoxon matched-pairs signed-rank test. ** $p < 0.05$.
